# Mobile Phone Based Strategies for Preconception Education in Rural Africa

**DOI:** 10.5334/aogh.2566

**Published:** 2019-07-09

**Authors:** Zemenu Yohannes Kassa, Zelalem Tenaw, Ayalew Astatkie, Melese Siyoum, Gezahegn Bekele, Kefyalew Taye, Shewangizaw Mekonnen, Zerai Kassaye

**Affiliations:** 1 School of Nursing and Midwifery, College of Medicine and Health Sciences, Hawassa University, Hawassa, ET; 2 School of Public Health, College of Medicine and Health Sciences, Hawassa University, Hawassa, ET; 3 School of Medicine, College of Medicine and Health Sciences, Hawassa University, Hawassa, ET

## Abstract

**Background:**

Prepregnancy health care is vital to alleviate and prevent maternal and neonatal disability and death.

**Objectives:**

The purpose of the study was to measure the levels of knowledge and attitude on preconception care and their determinants among women who delivered at government hospitals in a rural setting in southern Ethiopia.

**Methods:**

A facility-based cross sectional study was done from January 01 to February 30, 2017 on a sample of 370 women who delivered at government hospitals in Wolayita zone. The mothers were selected using systematic random sampling technique. The data were collected using structured and pretested interviewer administered questionnaires at the postnatal ward of each hospital. Data were analyzed using bivariate and multivariable techniques.

**Results:**

The result showed that 53% (95% confidence interval [CI]: 47.8%, 58.1%) of mothers who delivered at public hospitals had adequate level of knowledge on preconception care, whereas 54.3% (95% CI: 49.2%, 59.5%) possessed positive attitude to preconception care. Mothers who have radio, planned pregnancy and have participated in community meetings related to preconception care had a meaningfully higher odds of good level of knowledge to preconception care. Ordinal regression showed that women who own mobile phone had at least three times significantly higher odds of positive attitude to preconception care, whereas women who have participated community meetings had lower odds of positive attitude on preconception care.

**Conclusions:**

The results revealed that the levels of mothers’ knowledge and positive attitude on preconception care are low relative to other studies. Using transistor radio and mobile phone have significant effect in improving the knowledge and attitude of reproductive age women on preconception care. Hence, providing community health education based on radio and/or mobile phone messaging could be useful in positively influencing the knowledge and attitude of women on preconception care.

## Background

Preconception health care is a set of prepregnancy interventions to reduce the influence of biomedical, behavioral and social risks of mothers’ health, and unborn child health [1]. It can improve maternal and neonatal outcome by identifying, modifying bad habits and behaviors before conception and decreasing unintended pregnancies [2]. Besides, most of pregnancy and childbirth complications can be alleviated by implementation of preconception care at health institution, meanwhile in low resource settings preconception care is not regularly implemented [3].

Though both governments and civil societies in developing counties frontline agenda is maternal and neonatal health service, newborn and child death and stillbirth, of which 77% are preventable by creating platform for essential packages at community, health center and hospital levels have not yet been reduced to the expected level [4].

Worldwide, 216 maternal deaths occurred per 100,000 live births in 2015, of which 99% occurred in resource-constrained areas, especially south Asia and sub-Saharan Africa. The most substantial cause of mortality in women are: obstetric hemorrhage, preexisting medical conditions, hypertensive disease of pregnancy, infections/sepsis, unsafe abortion, and other indirect causes [4]. Globally, 2.6 million children died in the first month of life and neonatal mortality was estimated at 19 deaths per 1,000 live births [5]. The under-five mortality rate in 2015 was 42.5 per 1,000 live births [6,7]. In Ethiopia the maternal mortality rate is estimated at 412 per 100,000 live births in 2016, neonatal mortality at 29 deaths per 1000 live births, infant mortality at 48 deaths per 1,000 live births and the under-five mortality rate is estimated at 67 per 1000 live birth in 2016 [8].

Reproductive planning through preconception care could reduce 71% of unwanted pregnancies, thereby eliminating 22 million unplanned births, 25 million induced abortions and 7 million miscarriages [9,10]. Similarly, lack of preconception care and low folic acid supplementation for women in developing countries might increase the risk of neural tube defect in newborns by four times, compared with developed countries [11].

The basic concept of preconception care is to advise women of childbearing age away from any negative health behaviors or conditions that might affect a future pregnancy [12]. “A reproductive health plan reflects a person’s intentions regarding the number and timing of pregnancies in the context of their personal values and life goals.” This health plan will increase the number of planned pregnancies and encouraged persons to address risk behaviors before conception, reducing the risk of adverse outcomes for both the mother and unborn child [13,14].

A study done in Kelantan, Malaysia found that 51.9% of women attending maternal health clinic had good level of knowledge on preconception care and 98.5% had positive attitude regarding preconception care [15]. A study done in Egypt revealed that 39.2% of pregnant women attending ANC at Ain Shams University Hospital knew about the role of folic acid supplementation in prevention of congenital anomalies [16]. A community-based study done in Ethiopia revealed that 27.5% of reproductive age women had good level of knowledge regarding preconception care [17].

Studies suggested antenatal care ought to initiate before pregnancy to improve pregnancy outcome. Implementation of preconception care in maternity care unit is crucial to achieve the sustainable development goal (SDG) targets in relation to maternal, neonatal and child health, by decision makers and stakeholders. However, evidence on the levels of knowledge and attitude toward preconception care amongst women in rural African settings is scarce. The purpose of the study was therefore to measure the levels of knowledge and attitude on preconception care and their determinants among women who delivered at government hospitals in a rural setting in southern Ethiopia.

## Methods

### Study design and setting

A hospital-based cross-sectional study was done from January 1 to February 30, 2017, among mothers who delivered in public hospitals in Wolayita Zone and who were on immediate postnatal ward. Wolayita zone is found in the Southern Nations, Nationalities and Peoples Regional State of Ethiopia. According to the 2007 census of Ethiopia, the total population of the zone was 1.7 million. The public health institutions found in the zone were one referral hospital, four district hospitals and 70 health centers (5 urban and 65 rural). The total number of births from the five hospitals in 2016 was 7445 (Otona Hospital 3511, Bonbe hospital 1228, Halale hospital 1142, Bitana Hospital 956, and Bale Hospital 608).

### Study population and sampling procedures

Study populations were women who delivered at government hospitals in the Wolayita zone during the study period. Mothers who had loss of consciousness, had mental problem, and were referred to other hospitals were excluded.

Sample size was determined using the software Epi Info version 7 with the following assumptions: 95% confidence interval, an anticipated proportion of knowledge of preconception care of 10.4% based on a study in Nigeria [18], 4% of margin of error and a design effect of 1.5. The calculated sample size was 336. Combined with the 10% non-response rate, total sample was 374.

All public hospitals in the Wolayita zone were included in the study, and the sample size was proportionally allocated into five public hospitals based on number of deliveries each hospital. Systematic random sampling procedure was used to select study participants in each hospital. Monthly expected number of deliveries at public hospitals in Wolayita zone was 620; thus the sampling interval used was 2.

The questionnaires were prepared by reviewing the existing literatures. The questionnaire was prepared in English and then translated to Wolaytigna, and back to English to check uniformity. The questionnaire consisted of 57 items: 13 sociodemographic items, 6 obstetric items, 4 source of information items, 23 knowledge variables, and 11 attitude items. For attitude items, the Likert scale was used (1-strongly disagree, 2-disagree, 3-neutral, 4-agree and 5-strongly agree). During analysis, the Likert scale items were categorized into three response categories to compute women’s attitude on preconception care: disagree (by merging 1-strongly disagree and 2-disagree), neutral and agree (by merging 4-agree and 5-strongly agree).

In Hawassa University Comprehensive Specialized Hospital, a pretest was carried out with 5% of study participants. Based on the pretest findings, amendment was done before initiation of actual data collection.

Data were collected using structured and pretested interviewer administered questionnaire through face-to-face by 10 midwives who had received training on basic emergency obstetrics and newborn care (BEmONC) and who can fluently communicate in the local language (Wolaytigna). Training was given to data collectors for three days on data collection methodology and related issues prior to the start of data collection time and were closely supervised during the data collection period.

### Statistical analysis

Data entry was done EPI Data 3.1 and transferred to SPSS version 20.0 for analysis. Based on 23 knowledge items, we computed an overall knowledge score for each study participant. Those who had knowledge score above the mean knowledge score were level as “adequate knowledge” whereas at or below the mean knowledge score were categorized as “inadequate knowledge”. Eleven attitude items were recorded into disagree, neutral and agree. Those whose response was “agree” were considered as having “positive attitude” towards preconception care, whereas those whose response was “disagree” were regarded as having “negative attitude” towards preconception care; those with a “neutral” response were considered as having “neither negative nor positive attitude”. Descriptive analysis was done to calculate and describe the basic characteristics of the study participants knowledge and attitude to preconception care. Binary logistic regression was used to identify the correlates of knowledge on preconception care, while ordinal regression was used to identify correlates of attitude towards preconception care. Adjusted odds ratios (AORs) with 95% confidence intervals (CIs) were used to judge the presence and strength of association between dependent and independent variables. A P value of <0.05 was taken as statistically significant.

## Results

### Socio-demographic characteristic of study participants

Three hundred seventy women participated in this study with a 99% response rate. The participants’ ages ranged from 38 to 50, with a mean age of 25 (±4) years. Wolayita was the dominant ethnic group (91.9%). Three hundred sixty three (98.1%) were married. The majority (69.7%) of the participants were housewives and 34.9% had completed primary school (Table 1).

**Table 1:** Socio-demographic characteristics of women who gave birth at government hospitals in the Wolayta zone, South Ethiopia, February 2017.

Variables	(n = 370)	Frequency	Percentage

**Age**	15–19	26	7
	20–24	122	33
	25–29	149	40.3
	30–34	55	14.9
	35–38	18	4.9
**Religion**	Orthodox	112	30.3
	Muslim	10	2.7
	Protestant	238	64.3
	Catholic	8	2.2
	Jehovah witness	2	0.5
**Ethnicity**	Wolayita	340	91.9
	Amara	6	1.6
	Oromo	5	1.4
	Gamo	12	3.2
	Others®	7	1.9
**Marital status**	Married	363	98.1
	Single	5	1.4
	Widowed	2	0.5
**Occupation of The mother**	House wife	258	69.7
	Government employed	34	9.2
	Private employed	13	3.5
	Merchant	54	14.6
	Daily labor	7	1.9
	Farmer	4	1.1
**Occupation of spouse**	Farmer	123	33.2
	Government employed	80	21.6
	Private employed	45	12.2
	Daily labor	14	3.8
	Merchant	103	27.8
	Other	5	1.4
**Residency**	Urban	162	43.8
	Rural	208	56.2
**Monthly income**	<1313Ethiobirr (<59.7USD)	198	53.5
	<1313Ethiobirr (<59.7USD)	172	46.5
**Family size**	1–2	63	12.4
	3–5	369	72.4
	6–9	78	15.3
**Educational status of woman**	Informal education	110	29.7
	Primary school complete	151	40.8
	Secondary school and above	109	29.5
**Educational status of spouse**	Informal education	53	14.3
	Primary school complete	129	43
	Secondary school and above	158	42.7
**Communication**	Have radio	248	67
	Have Television	100	27
	Have Mobile	202	54.6
	Have health care providers as a relative	136	36.8
	Have regular community meeting regarding maternal health	89	24.1
	Have meeting with health extension worker	181	48.9
	Have health care providers as a friend	109	29.5
**Time taken to reach health institution**	<30 minutes	205	55.4
	>30 minutes	165	44.6

Others®-Dawro, Hadya, Sltie, Gurage.

* 1 USD was 22 Ethiopian birr.

Income under extreme poverty <$1.25 USD per day.

### Obstetric characteristics of study participants

In 296 (80%) of the mothers, the recent pregnancy was planned. Nearly two-thirds (65.1%) of mothers had used family planning before the current pregnancy. Ninety-eight (26.5%) of the mothers were primigravidae and 272(73.5%) were multigravidae, whereas 110 (29.7%) were primipara and 260 (70.3%) were multipara. Two hundred eighty-three (76.5%) of the participants had antenatal contact for this pregnancy, of whom 152 (41.1%) had four or more ANC contacts (Table 2).

**Table 2:** Obstetric history of women who delivered at government hospitals in the Wolayita zone, South Ethiopia, February 2017.

Variables	(N = 370)	Frequency	Percentage (%)

**Have family planning use history**	Yes	241	65.1
	No	129	34.9
**Gravida**	Prim gravida	98	26.5
	Multigravida	272	73.5
**Parity**	Primipara	110	29.7
	Multipara	260	70.3
**Is pregnancy plan**	Yes	296	80
	No	74	20
**ANC follow up**	Yes	283	76.5
	No	87	23.5
**Number of ANC visit**	No visit	20	5.4
	1	9	2.4
	2	44	11.9
	3	130	35.1
	4	152	41.1
	More than four	15	4.1

### Level of mothers’ knowledge of preconception care

**Figure 1:**
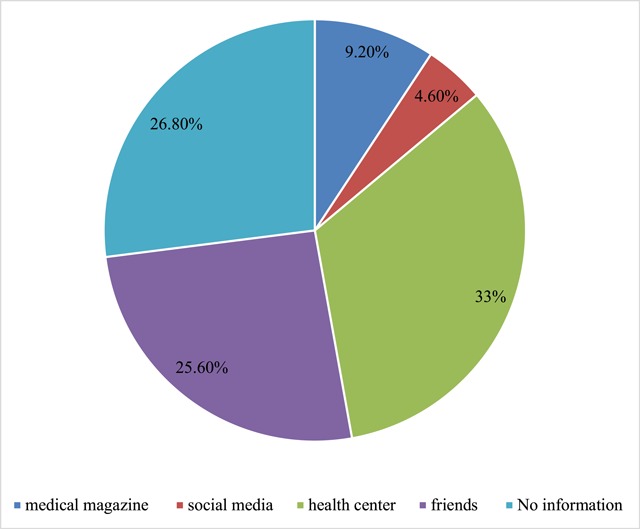
Source of information regarding preconception care amongst women who delivered at government hospitals in Wolayita Zone, South Ethiopia, February 2017.

The lowest and highest knowledge scores of the mothers were zero to twenty three. One hundred ninety-six (53%) (95% CI: 47.8%, 58.1%) of women had adequate level of knowledge of preconception care (Table 3). The main source of information were health institutions (33%) and friends (26.5%) (Figure 1).

**Table 3:** Women’s knowledge of preconception care who delivered at government hospitals in the Wolayita zone, South Ethiopia, February 2017.

Variable	(N = 370)	Frequency	Percent

**Avoid bad habits when planned to pregnancy**	Yes	311	84.1
	No	59	15.9
**Adjust their life when planned to pregnancy**	Yes	324	87.6
	No	46	12.4
**Avoid smoking when planned to pregnancy**	Yes	281	75.9
	No	89	24.1
**Avoid drinking alcohol when planned to pregnancy**	Yes	291	78.6
	No	79	21.4
**Avoid multiple sexual partners when planned to pregnancy**	Yes	303	81.9
	No	67	18.1
**Test HIV/AIDS when planned to pregnancy**	Yes	302	81.6
	No	68	18.4
**Take folic acid and multivitamins to prevent neural tube defects**	Yes	210	56.8
	No	160	43.2
**Take iron sulfate to prevent anemia?**	Yes	293	79.2
	No	77	20.8
**Avoid illicit drugs when planned to pregnancy**	Yes	262	70.8
	No	108	29.2
**Stop over exercising when planned to pregnancy**	Yes	287	77.6
	No	83	22.4
**Stop caffeine drinking when planned to pregnancy**	Yes	110	29.7
	No	260	70.3
**Stop mercury from consumption of seafood when planned to pregnancy**	Yes	99	25.9
	No	274	74.1
**Away from Pesticides/insecticides chemicals when planned to pregnancy**	Yes	217	58.6
	No	153	41.4
**Away from contact with substances like lead in paints when planned to pregnancy**	Yes	102	27.6
	No	268	72.4
**Away from exposure to occupational hazards when planned to pregnancy**	Yes	281	75.9
	No	89	24.1
**Maintain body weight when planned to pregnancy**	Yes	241	65.1
	No	129	34.9
**Take balance diet when planned to pregnancy**	Yes	266	71.9
	No	104	28.1
**Check STI when planned to pregnancy**	Yes	301	81.4
	No	69	18.6
**Take ordinary multivitamins when planned to pregnancy**	Yes	257	69.5
	No	113	30.5
**Take ordinary vitamin D when planned to pregnancy**	Yes	112	30.3
	No	258	69.7
**Take omega 3 vitamins when planned to pregnancy**	Yes	18	4.9
	No	352	95.1
**Take ordinary zinc when planned to pregnancy**	Yes	18	4.9
	No	352	95.1
**Street drugs when planned to pregnancy**	Yes	242	65.4
	No	128	34.6

### Women’s attitude regarding preconception care

Among the total of 370 respondents, 300 (81.1%) of the mothers agreed that a hospital setting is the best place to provide preconception care and 277 (74.9%) of women also agreed that preconception care is an important health issue for women of childbearing age. However, 54 (14.6%) of women agreed that there is not enough time to plan to get preconception care. Overall, 201 (54.3%) (95% CI: 49.2%, 59.5%) of mothers had positive attitudes towards preconception care, 23 (6.2%) (95% CI: 4.1%, 8.9%) of mothers had neither positive nor negative (neutral) attitudes towards preconception care and 146 (39.5%) (95% CI: 34.6%, 44.6%) of mothers had negative attitudes towards preconception care (Table 4).

**Table 4:** Women’s attitude on preconception care who delivered at government hospitals in Wolayita Zone, South Ethiopia, February 2017.

Parameter (N = 370)	SA&A		Neutral		SD&D	

	N	%	N	%	N	%

**Preconception care does not have any effect on pregnancy outcome**	160	43.2	53	14.3	157	42.4
**Preconception care is an important health issue for women of child bearing age**	277	74.9	57	15.4	36	9.7
**A dedicated clinic for preconception care is a luxury service**	209	56.5	56	15.1	105	28.4
**A hospital setting is the best place to provide preconception care**	300	81.1	33	8.9	37	10
**Preconception care is a high priority all mother to plan pregnancy**	241	65.1	74	20	55	14.9
**I am not the most suitable person plan to get preconception care**	69	18.6	40	10.8	261	70.5
**There is not enough time to plan to get a preconception care**	54	14.6	43	11.6	273	73.8
**Health institutions exercise preconception care**	96	25.9	41	11.1	233	63
**Do you think high-risk mothers only start preconception care when planned to pregnancy?**	106	28.6	28	7.6	236	63.8
**History congenital anomalies only use preconception care**	113	30.5	33	8.9	224	60.5
**Preconception care depends on health care providers’ willingness**	262	70.8	46	12.4	62	16.8

SA: Strongly agree, A: agree, SD: strongly disagree and D: disagree.

### Determinants of knowledge and attitude regarding preconception care

Study participants who had radio (AOR: 2.91; 95% CI: 1.69, 5.43), planned pregnancy counterpart (AOR: 5.76; 95% CI: 2.84, 11.67), and had participated in community meetings related to preconception care (AOR: 2.96; 95% CI: 1.62, 5.43) had significantly higher odds of a good level of knowledge of preconception care (Table 5).

**Table 5:** Determinants of knowledge of preconception care amongst women who delivered at government hospitals in the Wolayita zone, South Ethiopia, February 2017.

Variable	Knowledgeable (N = 196)	Not knowledgeable (N = 174)	COR 95% CI	AOR 95% CI

**Do you have a radio?**				
Yes	159 (43)	89 (24.1)	4.10 (2.58, 6.54)*	2.91 (1.69, 5.43)*
No	37 (10)	85 (23.4)	1	1
**Do have health care providers as relatives?**				
Yes	88 (23.8)	48 (13)	2.13 (1.384, 3.306)*	1.29 (0.74, 2.26)
No	108 (29.2)	126 (34.1)	1	
**Is the pregnancy planned?**				
Yes	183 (49.5)	113 (30.5)	7.60 (3.995,14.455)*	5.76 (2.84, 11.67)*
No	13 (3.5)	61 (16.5)	1	1
**Do you have community meetings related to preconception care?**				
Yes	67 (18.1)	22 (5.9)	3.588 (2.100, 6.132)*	2.96(1.62, 5.43)*
No	129 (34.9)	152 (41.1)	1	1
**Do you have health care providers as friends?**				
Yes	75 (20.3)	34 (9.2)	2.552 (1.591, 4.094)*	1.36 (0.74, 2.47)
No	127 (34.3)	140 (37.8)	1	
**Educational status of spouse**				
Informal education	14 (3.8)	39 (10.5)	0.301 (0.151, 0.597)	
Primary school	96 (25.9)	63 (17)	1.28 (0.817, 1.993)*	1.31 (0.73, 2.36)
Secondary and above	86 (23.2)	72 (19.5)	1	1

* P < 0.05.

On the other hand, multivariable ordinal regression showed that women who had mobile phone had a twofold higher chance of a positive attitude (AOR: 2.17, 95% CI: 1.31, 3.59) and those who had participated in community meetings related to preconception care had decreased odds of a positive attitude towards preconception care (AOR: 0.36, 95% CI: 0.22, 0.60) (Table 6).

**Table 6:** Determinants of attitude to preconception care amongst women who delivered at government hospitals in the Wolayita zone, South Ethiopia, February 2017.

Variable	Attitude			COR 95% CI	AOR 95% CI

	Disagree (N = 146)	Neutral (N = 23)	Agree (N = 201)		

**Residency**	Rural		1.94 (1.29, 2.93)*		1.49 (0.91, 2.44)
	Urban		1		1
**Mobile phone**	Yes		2.29 (1.52, 3.44)*		2.17 (1.31, 3.59)*
	No		1		1
**Do you have community meetings related to maternal health?**	Yes		0.35 (0.22, 0.57)*		0.36 (0.22, 0.60)*
	No		1		1
**Spouse education**	Informal education		0.58 (0.38. 0.90)*		1.32 (0.63, 2.76)
	Primary school complete		0.82 (0.44,1.52)*		0.8 (0.48, 1.34)
	Secondary school and above		1		1

* P < 0.05.

## Discussion

Findings revealed that level of knowledge of preconception care amongst women who delivered at government hospitals in the Wolayita zone is 53%. This finding is inconsistent with the findings in Northwest Ethiopia (27.5%) [17], Sudan (11.1%) [19], Nigeria (2.5%) [9], Iran (10.4%) [20], Saudi Arabia (37.9%) [21], United Arab Emirates (46.4%) [22], and Turkey (46.3%) [23]. The possible explanation for higher level of knowledge in the present study could be the time of study, maternal health is given high attention which may result in an overall increase in knowledge of issues related to maternal health. Contextual differences in the study settings could also account for the observed differences.

On the other hand, it is consistent with studies done in Malaysia (51.9%) [15], and in Qatar (53.7%) [24]. However, this fining is lower than the study done in Canada (70%) [25], Jordan (85%) [26], British Colombia (71%) [27], Saudi Arabia (84.6%) [28], and in the United States of America (76%) [29]. The possible explanation could be low level of knowledge due to health sector infrastructure difference, socioeconomic difference, lack of health wellness clinic in the area of the present study, lack of preconception service across Ethiopia, lack of promotion of preconception care by mass media, and low commitment of health care providers due to high load of clients.

In this study the correlates of knowledge of preconception care were found to be possession of transistor radio, planned pregnancy, and having participated in community meetings related to preconception care. Women who had a radio had were three times more likely to have adequate knowledge of preconception care. This is inconsistent with studies done in Ethiopia and Nigeria [9,17]. The higher level of knowledge of preconception care amongst women who possess a transistor radio and who participate in community meetings related to preconception care can be due to exposure of such mothers to health information via radio and also during community meetings. The community meetings could also create a platform for women to share their positive and negative childbirth experiences and prevention mechanisms. Similarly, women who planned the recent pregnancy were six times more likely to have adequate knowledge of preconception care, which coincides with the findings in Brazil [30]. The possible explanation could be reproductive age women who planned pregnancy are expected to know their healthiness correlated to maternal health care and may thus have also a better awareness of issues correlated to preconception care.

In this study 54.3% of mothers were found to have positive attitude towards preconception care. This finding is incomparable with studies done Malaysia (98.5%) [15] and USA (98%) [29]. The difference might be due availability and accessibility of the service in settings with better socioeconomic status such as in Kelantan, Malaysia and USA.

Women who possess mobile cell phones are more than twice as likely to have positive attitudes towards preconception care; however, women who have participated in community meetings related to preconception care had decreased odds of positive attitudes towards preconception care. The reason women who possess cell phone have higher odds of positive attitudes towards preconception care could be due to better exposure of such women to health information via frequency modulated (FM) radio services, which are available in most cell phones and for some of the literate mothers via mobile internet. Women who posses mobile phones may also generally be in a better socioeconomic position and hence may have more positive attitudes to health care services. The reason women who participate in community meetings have decreased odds of positive attitudes is difficult to explain, but this could be a result of being fed up with regular participation in community meetings.

The strength of this study relative to previous studies is incorporating relevant variables that were not addressed previously, such as having planned pregnancy, possession of transistor radio and participating in community meetings related to preconception care. The limitation of this study is that it did not incorporate both sides, such as partners of women. Outcomes can be, to some degree, affected by recall and social desirability biases.

## Conclusion

Levels of women’s knowledge and positive attitude of preconception care among women who delivered at government hospitals in rural southern Ethiopia is low compared with other studies. Using a transistor radio and mobile phone have significant effects in improving the knowledge and attitude of reproductive age women on preconception care. Hence, providing community health education based on radio and/or mobile phone messaging could be useful in positively influencing the knowledge and attitude of women on preconception care.

## Date Accessibility Statement

All data on which this article is based are included within the article.

## Abbreviations

AOR: Adjusted Odds Ratio

BEmONC: Basic Emergency Obstetrics and Newborn Care

CI: Confidence Interval

SDG: Sustainable Development Goals

SPSS: Statistical Package for Social Sciences
